# ZAMSTAR, The Zambia South Africa TB and HIV Reduction study: Design of a 2 × 2 factorial community randomized trial

**DOI:** 10.1186/1745-6215-9-63

**Published:** 2008-11-07

**Authors:** Helen M Ayles, Charalambos Sismanidis, Nulda Beyers, Richard J Hayes, Peter Godfrey-Faussett

**Affiliations:** 1 Department of Infectious and Tropical Diseases, London School of Hygiene & Tropical Medicine, London, UK; 2 ZAMBART Project, University of Zambia, Lusaka, Zambia, Africa; 3 Department of Epidemiology and Population Health, London School of Hygiene & Tropical Medicine, London, UK; 4 Desmond Tutu TB Centre, Stellenbosch University, Tygerberg, South Africa

## Abstract

**Background:**

TB and HIV form a deadly synergy in much of the developing world, especially Africa. Interventions to reduce the impact of these diseases at community level are urgently needed. This paper presents the design of a community randomised trial to evaluate the impact of two complex interventions on the prevalence of tuberculosis (TB) in high HIV prevalence settings in Zambia and South Africa.

**Methods:**

The interaction between TB and HIV is reviewed and possible interventions that could reduce the prevalence of TB in HIV-endemic populations are discussed. Two of these interventions are described in detail and the design of a 2 × 2 factorial community randomised trial to test these interventions is presented. The limitations and challenges of the design are identified and discussed.

**Conclusion:**

There is an urgent need to reduce the prevalence of TB in communities highly affected by HIV. Potential interventions are complex and require innovative trial designs to provide the rigorous evidence needed to inform health policy makers and to ensure that resources are used optimally.

**Trial Registration:**

Number: ISRCTN36729271

## Background

Tuberculosis (TB) incidence is increasing in much of sub-Saharan Africa, largely due to the high prevalence of HIV infection. International TB control strategies rely on self-presentation of cases to the health services, diagnosis by sputum smear microscopy and the use of a 6–8 month course of multi-drug therapy to cure the disease[1]. However these strategies are currently failing to control TB in high HIV prevalence settings, due both to the massive burden of disease and the inadequacy of existing health systems to find and cure infectious cases coupled with the continuing stigma and denial that surround HIV and TB[2].

The Zambia/South Africa TB and AIDS Reduction (ZAMSTAR) study aims to use existing tools for diagnosis and treatment but to test new approaches to the delivery of TB control strategies to determine whether these can reduce the prevalence of TB and HIV at community level.

### Rationale for ZAMSTAR

The first mechanism by which prevalence of TB could be reduced is through more efficient detection and treatment of infectious cases of TB. Prior to the HIV epidemic, a large body of work provided the backbone for the DOTS strategy, which uses passive case-finding to detect TB cases[3]. Recent work highlights the importance of case-finding and the need to adapt the existing approach to incorporate a more aggressive form of case-detection. The challenge in poorly-resourced settings is how to achieve this without overburdening an already overstretched health service. Sustainable case-finding will need to involve collaboration between the formal health services, other government sectors, community based organisations and the community.

Reducing the reactivation of latent TB infection by preventive therapy and improving the immune function of HIV-infected individuals is another mechanism that could reduce TB prevalence. Randomised trials in developing countries have demonstrated the efficacy of isoniazid in preventing TB in HIV-positive subjects at the individual level[4], but its population-level effectiveness as a TB control measure in HIV prevalent settings has not been proven. Feasibility studies have demonstrated that the main barriers to the successful implementation of this intervention are the difficulties in excluding active TB and low adherence to the course of treatment[5]. For the intervention to be effective at community level, uptake must be increased. Uptake is currently hampered by lack of knowledge of HIV status and poor access to counselling, testing and care as well as by poverty and rural deprivation. TB preventive therapy is also recommended for the household contacts of cases of infectious TB but in most countries is not implemented due to the low priority given to this intervention, lack of available health workers and limited availability of drugs.

HIV infection is widely acknowledged as the driving force behind the current TB epidemic in Southern Africa. Long term control of TB must include addressing the problem of HIV prevention. Proven interventions to reduce the incidence of HIV at a community level are disappointingly few. Provision of voluntary counselling and testing (VCT) has been promoted as an important step in supporting individuals to change behaviour by facilitating knowledge of HIV status. Few trials have used HIV incidence as an end point, although a recent study in Harare among factory workers showed that despite good uptake of VCT HIV incidence was not reduced[6]. Other studies have shown some evidence of behaviour change in HIV-positive individuals but little change has been seen in HIV-negative ones[7]. VCT has been shown to be most effective when offered to couples, since this allows discordant couples to make realistic plans to reduce the risk of HIV transmission[8,9]. Antiretroviral drugs are rapidly becoming more available in developing countries and it is hoped that the increased availability of these drugs may increase uptake of HIV testing and counselling and also reduce transmission of HIV by reducing viral load. This theory is unproven, however, and in wealthier countries, already using antiretrovirals, there is evidence that sexual behaviour has become more, rather than less, risky since their use became widespread and that HIV incidence may be increasing[10].

Finally, stigma is recognised as a barrier for individuals to access diagnosis and care for both TB and HIV. Studies have demonstrated that stigma prevents people (including TB patients or suspects) from: accessing health care, learning their HIV status, disclosing their HIV status to partners, changing unsafe behaviour, and caring for people living with HIV/AIDS (PLWHA), and that the most extreme forms of stigma are often found in households and health facilities [11].

ZAMSTAR has addressed these issues by developing two interventions that combine all of the above strategies and that can be applied at community level. These interventions are community-based enhanced case finding for tuberculosis (ECF) and household counselling and provision of combined TB/HIV prevention services at the household level (HH). The effectiveness of these interventions at population level will be evaluated through a community-randomised trial with a 2 × 2 factorial design.

### Design

#### Study setting

**Figure 1:**
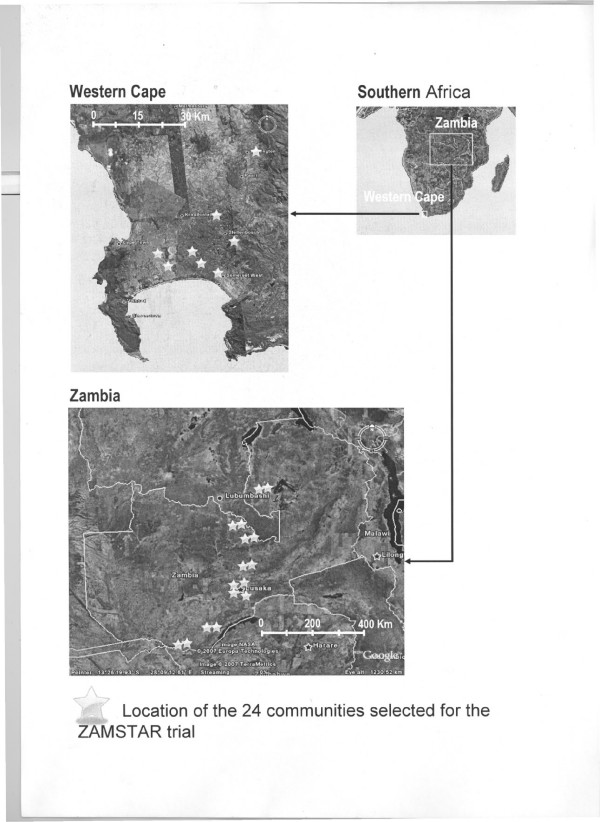
Map of ZAMSTAR communities.

The study is being conducted in Zambia and South Africa, two countries highly affected by the TB and HIV epidemics, and forms part of the CREATE consortium . Study communities have been selected based on TB notification rates greater than 400/100,000 per annum, high HIV seroprevalence and proximity to a TB diagnostic centre. The communities selected are in five provinces of Zambia and in Western Cape province of South Africa and include both urban and rural communities (Fig 1).

#### Design of the interventions

##### Enhanced case finding intervention (ECF)

Three components of ECF are being employed at the community and health centre levels.

##### Open laboratory access at health centre

Individuals are able to directly access sputum smear examination for TB at the health centre laboratories, without having to see a clinician or be referred for this service. This service is available continuously and is widely advertised in the community.

##### Community mobilisation and mobile sputum collection points

Community mobilisation is conducted to educate the community about TB and its diagnosis. Mobile sputum collection points are set up at strategic points in the community where symptomatic members of the public are asked to submit sputum samples on the spot and to return the following day for the results. The location of these sputum collection points ensure that all members of the community have access to sputum examination within 30 minutes walk of their home at least three times per year.

##### School mobilisation campaign

All schools in the community receive an annual education programme about HIV and TB. Children are encouraged to take messages about the transmission and diagnosis of TB home so that family members who have been coughing for more than three weeks can submit sputum samples at collection points set up in the schools or at the clinic.

Any cases of TB found by the ECF activities are treated via the routine clinic-based DOTS services.

### Household intervention (HH)

All TB cases diagnosed and notified through the TB control programme are invited to participate in this intervention. All households of TB patients receive information about HIV and TB within the home from a specially trained counsellor. The household members are encouraged to be tested for HIV and screened for TB. A "shared confidentiality" is encouraged between household members to facilitate open discussion of the outcome of the investigations. The household is also encouraged to take responsibility for the member diagnosed with TB and to supervise and encourage treatment compliance. Household members with HIV who do not have active tuberculosis diagnosed are offered TB preventive therapy, as are all children under the age of 6 years, in keeping with national policies (although these are otherwise poorly implemented). Adherence is supported within the family, as well as by the routine treatment support service of the district. All HIV-positive household members will be encouraged to obtain HIV care including antiretroviral therapy (ART) which is provided by the health centres or district hospitals in all of the communities.

### Standard TB/HIV control practice

In all arms of the study, standard TB control practices are supported and, where necessary, improved such that all diagnostic centres are applying a model DOTS programme. Integration of TB and HIV services will be facilitated including diagnostic counselling and testing for HIV in TB patients, referral networks to enable TB patients to access HIV care, screening of HIV positive individuals for TB and the provision of isoniazid preventive therapy for eligible individuals[12].

### Design of the randomised trial

The interventions are being evaluated by means of a 2 × 2 factorial community randomised trial.

The primary study questions are:

• Does enhanced tuberculosis case finding (ECF), by a strategy of community mobilisation and enhanced access to sputum microscopy, reduce prevalence of tuberculosis in the community?

• Does a strategy of combined TB/HIV activities at the household level (HH), that includes active case-finding, isoniazid preventive therapy, and psychosocial and adherence support for both treatment and prevention of tuberculosis and HIV, reduce prevalence of tuberculosis in the community?

The unit of randomisation will be the population accessing one TB diagnostic centre (which will be known as the "community"). A 2 × 2 factorial design will be used so that there will be four arms:

Arm 1: Standard TB/HIV Control activities at the clinic only

Arm 2: Standard TB/HIV Control activities at the clinic *plus *ECF

Arm 3: Standard TB/HIV Control activities at the clinic *plus *HH

Arm 4: Standard TB/HIV Control activities at the clinic *plus *ECF *plus *HH

The primary analysis will be a comparison of ECF Vs no ECF (arms 2 & 4 vs arms 1 & 3) and of HH Vs no HH (arms 3 & 4 vs arms 1 & 2). This allows the questions posed in the specific objectives to be addressed. The factorial design will also allow arm 4 to be compared directly to arm 1, referred to as the control arm in this paper, to address the question:

• What is the overall effect of the combined interventions (ECF+HH) on the prevalence of tuberculosis in the community?

A cohort of consecutive TB patients and their households in each community will be followed for the duration of the interventions to allow analysis of household-level outcomes, using both epidemiological and social science methods.

### Outcome measures

The primary outcome for the study is the prevalence of tuberculosis after three years of intervention, measured through surveys of a random sample of adults (aged > 15 years) from each community.

Secondary outcomes at community level are:

• Incidence of tuberculosis infection measured by repeat tuberculin skin testing (TST) in primary school children found to be TST negative at baseline.

• Tuberculosis treatment outcomes (Cure, Completed treatment, Failed, Defaulted, Died, Transferred) measured by cohort analysis of strengthened tuberculosis register data.

• Additional tuberculosis cases detected by ECF and HH measured using the strengthened laboratory register.

• Uptake of HIV testing and counselling and uptake of and adherence to tuberculosis preventive therapy measured using the testing and counselling register.

• Costs of each component of the intervention and cost-effectiveness comparisons of strategies to find and prevent cases of tuberculosis and HIV.

Secondary outcomes at household level, as measured on a cohort of households are:

• Cumulative HIV incidence measured by serial HIV testing among those over 15 years old.

• Uptake of HIV testing and counselling and uptake and adherence to tuberculosis preventive therapy among household members of TB patients

• Tuberculosis transmission within the household measured by TST and Quantiferon-Gold IT conversion rates

• Cumulative incidence of tuberculosis disease in household contacts

• Stigma levels measured using a quantitative stigma score

### Outcome measurement

#### Baseline assessment of communities

In order to stratify and restrict the randomisation of communities a baseline assessment was conducted by collating information on TB notification rates, TB treatment outcomes, HIV prevalence data and the prevalence of tuberculous infection in school children (see below). In addition to the quantitative baseline assessment, a rapid qualitative appraisal of the communities was conducted. This appraisal involved community mapping, observation and key informant interviews to understand the domains of TB in the local community including population movement, congregation points, beliefs and knowledge about TB and HIV as well as general socio-economic information about the communities

#### Tuberculin Skin Testing

Baseline measurement of tuberculous infection in all ZAMSTAR communities was estimated by means of tuberculin skin testing (TST) surveys among primary school children[13]. These community-wide surveys served three objectives: to characterise ZAMSTAR communities, with regards to TB infection, in relative terms; to inform the randomisation of the communities into the four intervention arms; and to provide data for one of the community-wide secondary outcomes. The design as well as results from these surveys will be described in a separate paper.

The 22,000 children involved in these surveys are being followed up for the three years of the intervention and will be retested at the end of the study to measure the incidence of TB infection in those who were uninfected at baseline.

#### TB prevalence surveys

The primary outcome of the study will be measured by conducting TB prevalence surveys on 5000 randomly selected members of each community. The methodology to be used for these surveys will involve TB culture of 1 respiratory specimen from each adult. The prevalence of TB will be measured as the proportion of these samples that grow *Mycobacterium tuberculosis *using a standard liquid culture technique (MGIT, Becton Dickinson).

#### Secondary outcome cohort (SOC)

150 consecutive TB patients and their households are being recruited in each community and asked to complete a baseline questionnaire containing demographic and specific TB and HIV data as well as questions about stigma relating to TB and HIV. Adults are asked to provide a serum sample to test for HIV and both adults and children are tested for TB infection. This cohort will be visited after eighteen months and again after three years of the intervention to allow measurement of HIV incidence, the incidence of TB infection, the incidence of TB disease and changes in perceptions of stigma.

#### TB/HIV indicators

In each community TB/HIV programme indicators as described in the WHO guide to Monitoring and Evaluation of TB/HIV collaborative Activities [14] will be collected throughout the study period. These will provide the secondary outcome measurements at community level.

### Sample size

**Figure 2:**
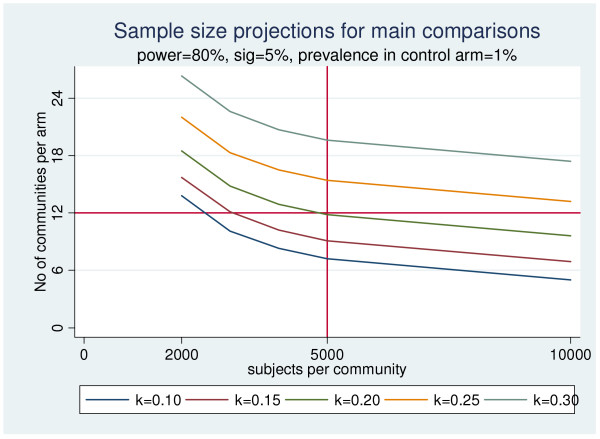
Graph showing the effects on sample size of different inter-cluster coefficients of variation (k).

Based on data from prevalence studies in Cape Town [15] and WHO estimates of tuberculosis prevalence[16] we assumed a prevalence of tuberculosis in the control arm of 1%. We powered our study in order to be able to detect a 30% reduction in prevalence of tuberculosis due to each intervention individually and a further 30% reduction in tuberculosis prevalence when the two interventions are combined. To achieve 80% power of detecting a significant difference (2-sided p < 0.05), an assumed between-community coefficient of variation *k *= 0.20 and 5,000 individuals sampled per community (Tab 1 and figure 2) we would need 12 communities in each arm. The total sample size will therefore be 24 communities, 6 in each of the four arms[17].

**Table 1:** Sample size calculations for power of 80% (two-sided, 5% significance level), based on sample of 5000 individuals per community and allowing for 20% losses, for each of the main comparisons (ECF vs non-ECF & HH vs non-HH)

Prevalence (%) in Control arm	Reduction in Prevalence due to each intervention (ECF or HH) individually	Prevalence (%) in ECF or HH arm	Overall number of communities needed (equally split into 4 arms)
**k = 0.15**			

0.6	30%	0.42	24
0.8	30%	0.56	20
1.0	30%	0.7	18
1.0	20%	0.8	41
1.0	10%	0.9	168

**k = 0.2**			

0.6	30%	0.42	30
0.8	30%	0.56	26
1.0	30%	0.7	24
1.0	20%	0.8	54
1.0	10%	0.9	227

**k = 0.25**			

0.6	30%	0.42	45
0.8	30%	0.56	42
1.0	30%	0.7	39
1.0	20%	0.8	93
1.0	10%	0.9	398

k = between-cluster coefficient of variation

The two primary study questions will be answered by comparing 12 communities with the other 12 (e.g. non-ECF arms 1 & 3 Vs. ECF arms 2 & 4 or non-HH arms 1 & 2 Vs. HH arms 3 & 4), allowing for the factorial design this means that we assume a TB prevalence of 1% in the control arm (arm1), 0.7% in single intervention arms (arm 2 and arm 3) and 0.49% in the arm with both interventions (arm 4). Overall, the primary study comparisons will therefore be able to detect the 30% reduction in TB prevalence from 0.85% to 0.595%.

For the secondary outcome of cumulative incidence of tuberculosis infection over three years of intervention, as measured by TSTs, we wish to detect a 30% reduction associated with either the ECF or HH intervention. The sample size is calculated to provide 80% power of detecting this difference (two-sided p < 0.05,) on the assumption that there will be 12 communities in each of the arms for the two main comparisons and with *k *= 0.20. Under these assumptions, and with an assumed 3% cumulative incidence of tuberculosis infection over three years (at 1% annual risk of infection) in the control arm and allowance for 20% loss to follow-up, we will need to initially recruit 800 children in each community.

HIV incidence will be measured in the secondary outcome cohort of 150 TB patients and their households per community. Each household would be expected to include 2–3 HIV negative adults at the start of the study. Allowing for loss to follow-up of 20%, this sample size will provide 80% power to detect a reduction in cumulative HIV incidence of 40%, (2-sided p <0.05)) assuming HIV incidence in those individuals not receiving the intervention is at least 0.7% per year (cumulative incidence 2.1% over 3 years).

### Randomization

Due to the small number of communities in the study we stratified randomisation by country and the prevalence of TB infection (as measured by baseline TST surveys and categorised as high/low within countries). We used restricted randomisation to ensure balance on TST prevalence, HIV prevalence, urban/rural, social context and geographical location. A two-stage public randomisation ceremony was conducted to select one of the possible allocations for the study arms and then to allocate the interventions to those study arms. The design and results of this allocation procedure have been described in another paper [18].

### Analytic plan

As a community randomised trial ZAMSTAR will be analysed both at the cluster level, by deriving summary statistics for each community, and at the individual level using data from each individual in each community. For these analyses statistical methods that take account of between- and within-cluster variation will be used. The analysis will give each community equal weight. There are three intervention comparisons that will be made assessing the effects of ECF alone (12 Vs 12 communities), HH alone (12 Vs 12 communities) and ECF plus HH combined (6 Vs 6 communities). Impact measures of the above comparisons will be based on ratios.

To account for the study design, suitable generalized linear models will be used, where appropriate, to allow for clustering by community. For the binary outcomes (prevalence, incidence) the overall risk/rate for each cluster will be calculated. Risks/rates for each cluster will be shown, by strata and arm. A log transformation to normalise data will be applied to the risk/rate for each cluster. The mean and SD of these log risks/rates will be used to obtain the geometric mean (if the log transformation is clearly inappropriate the arithmetic mean will be used instead) and associated 95% CI for each intervention arm of the study. Linear regression of the log mean risk/rate on strata and arm will be used to estimate the relative risk/rate and 95% CI associated with the intervention. The approximate variance for the mean risks/rates ratio is obtained based on the residual mean square from a two-way analysis of variance (ANOVA) of community log-risk on stratum and study arm. A 95% CI for the relative risk/rate will be calculated from this variance using a t-statistic with 16 degrees of freedom (df) for the two main comparisons and 10 df for the comparison of arm 4 Vs arm 1.

For the continuous outcomes such as intervention costs and stigma levels, the overall mean for each community will be calculated, and means for each community will be shown, by strata and arm. The arithmetic mean and SD of these mean scores/numbers and associated 95% CI for each intervention arm of the study will be calculated. Linear regression of the mean score/number on strata and arm (2-way ANOVA on intervention and strata) will be used to estimate the difference in score/number and 95% CI associated with the intervention.

Apart from calculation of crude effects, adjusted analyses to account for potential confounding effects will also be performed. Potential confounding factors will include age, sex, TB infection prevalence (as calculated by baseline TST surveys) as well as other factors found to differ between study arms at baseline and which may be risk factors for a particular outcome. For the binary outcomes (prevalence, incidence) logistic regression will be used to adjust for confounders at the individual level and cluster level, adopting a two-stage process. The regression model will include terms for the adjustment factors and strata, but not study arm. For each community the fitted model will be used to obtain the ratio of observed (O) to expected (E) or O/E events, and a log transformation applied to this ratio where appropriate. Linear regression of the log mean O/E on strata and arm (2-way ANOVA) will be used to estimate the risk/rate ratio and 95% CI associated with the intervention. The variance for the ratio of mean O/E is calculated from the residual mean square from an ANOVA of O/E on strata and arm.

For the continuous outcomes such as intervention costs and stigma levels, linear regression of the mean number/score will be used to adjust for confounders at the individual level. The regression model will include terms for the adjustment factors and strata, but not study arm. For each community the fitted model will be used to obtain the difference between observed and expected number of events. Linear regression of the difference O-E on strata and arm (2-way ANOVA) will be used to estimate the difference in (O-E) between arms and the 95% CI associated with the intervention. For all analyses, the robustness of the assumptions will be confirmed by a non-parametric stratified permutation test.

Analyses will also look at effects stratified by urban/rural classification, TB notification rates, ART usage, size of community and HIV prevalence. It is recognised that the study has low power to detect differences by subgroup and also the interaction between the interventions (ECF plus HH Vs standard TB/HIV control activities) and so the results of such analyses will be interpreted with caution.

### Ethical considerations

The research ethics committees of the University of Zambia, Stellenbosch University and the London School of Hygiene and Tropical Medicine have approved the study. Ongoing review and approval of the study will be maintained by these committees.

Community advisory boards representing community members have been established in all 24 communities. Permission for the study has also been obtained from national, provincial, district and local health and education authorities, community leaders and traditional leaders.

All individuals receiving the household intervention and all of those who participate in outcome measurement such as prevalence surveys, tuberculin surveys and secondary outcome cohort visits will also be asked to provide individual written informed consent.

A Data Safety and Monitoring Board has been established for all of the CREATE trials to monitor data from the trials. Regular trial monitoring is also carried out by the CREATE Consortium as study sponsor.

All communities will benefit from strengthened TB care and combined TB/HIV interventions that will ensure more linkages between the TB and HIV programmes and enable improved access to HIV care and especially ART. This study is embedded within the governmental health system in every health centre to ensure that these benefits are sustained.

## Discussion

ZAMSTAR is a trial of two complex public health interventions to assess whether either or both can reduce the prevalence of TB in high HIV prevalence settings. To test such complex interventions, especially when the disease targeted is infectious, requires the use of a community-randomised design. We anticipate that the interventions will act at the individual, household and community levels by finding more cases of tuberculosis more quickly and that this will firstly reduce transmission of tuberculosis in the household and the community and ultimately the prevalence of infection and disease. To enable us to capture all of these steps in the process requires that we also measure the outcomes for individuals, households and communities. We hope that by addressing the two diseases, TB and HIV, together we will also have an impact on HIV. We anticipate that our interventions will allow more individuals to learn their HIV status and to either protect themselves from acquiring HIV or to access necessary HIV care. We do not believe, however, that these interventions will be able to significantly reduce HIV incidence or prevalence at the community level but do hope to be able to show a reduction in incidence at the household level.

To increase trial efficiency, clusters in community randomised trials should be numerous and small[19]. In the context of the ZAMSTAR trial, however, we needed to balance this requirement with knowledge about the transmission of tuberculosis within populations. We believe that to measure effects on transmission and prevalence requires the use of large clusters that encompass entire and discrete populations, so that we can be fairly confident that any case of incident TB was caused by transmission from someone else in that community who had access to the intervention. Due to the necessity to embed the interventions within the existing health services, this dictated that we use the entire population attending one health centre as our community. Consequently although we only have 24 communities, they have a combined population of approximately 1.2 million. A 2 × 2 factorial design allows us to evaluate the two interventions at the same time, thus maximising the cost-efficiency of conducting such a large trial.

Contamination of a community by a neighbouring community with a different set of interventions was a concern and so the communities needed to be geographically and culturally distinct. Mobility into and out of the communities may also dilute the effect of the interventions and we tried to avoid communities that had high levels of mobility and are using census and qualitative studies to measure the degree of mobility. Additionally we needed to use communities that had high baseline levels of disease to allow us to measure the impact of the interventions, but we wished to maintain a range of urban and rural communities to retain generalisability. Combining these requirements has resulted in widely-spaced communities, using seven different languages and with many different cultures. Health and other infrastructures in these communities are generally poor, generating logistical challenges to conducting the study. Gaining community trust is a process that requires perseverance and understanding on the part of both researchers and community members. Without trust there will not be widespread community involvement in the study, which is necessary to ensure that the interventions work well.

As well as the logistical and field work challenges raised by such a study, there are statistical and design challenges. While the factorial design is cost-efficient, allowing us to evaluate the impact of two interventions in one trial [20], the primary analysis will be based on comparisons of the 12 communities with an intervention vs the 12 without this intervention. We are also powered to measure the combined effect of the two interventions compared with the control arm [arm 1 with arm 4], but not to compare the interventions with each other. A specific consideration in the analysis of factorial trials is the effect of interaction between the interventions and whether this can be accurately measured[21]. It is possible that the ECF intervention will have a larger effect on the primary outcome when combined with the HH intervention, and similarly the HH intervention will be applied to more households if more cases of TB are found using ECF. We do not believe that there will be a significant negative interaction between the two interventions and consider that both interventions will have independent effects. We acknowledge, however, that we will have limited power to detect interactions between the interventions.

The small sample size also affected our choice of randomisation strategies. Stratification and restricted randomisation were necessary to ensure balance between the arms of the trial and to reduce the between-community coefficient of variation[22].

Despite these challenges the study is underway and the interventions appear to be feasible and popular. One of the study's strengths is that it is embedded within the existing health services, working closely with district and provincial health teams and with clinic staff. The variety of cultures and infrastructure ensures that the results will be generalisable and we can already see that the interventions could be applied in the routine health setting. The primary outcome of the study, TB prevalence, will not be measured until 2010, but already important knowledge is being generated that is helping us understand the complex epidemiology of the TB/HIV epidemic. The results of this trial will provide much needed evidence for health policy makers as they strive to control TB and HIV.

## Abbreviations

ANOVA: Analysis of variance; ART: Anti-retroviral therapy; CI: Confidence Interval; DOTS: The international standard for TB control, that consists of a 5 point plan of political commitment, microbiological diagnosis, standardised drug regimens, uninterrupted drug supply and outcome monitoring. (Direct observation of therapy, short course); E: Expected; ECF: Enhanced case finding for TB; HH: Household level TB/HIV intervention; HIV: Human Immunodeficiency Virus; MGIT: Mycobacterial Growth Indicator Tube (Becton-Dickenson); O: Observed; PLWHA: People living with HIV/AIDS; SD: Standard Deviation; TST: Tuberculin skin test; VCT: Voluntary Counselling and Testing; WHO: World Health Organisation.

## Competing interests

The authors declare that they have no competing interests.

## Authors' contributions

HA originally conceived and designed the trial with PGF and wrote the drafts of the paper. CS helped to design the statistical framework of the trial and contributed to all drafts of the paper. NB helped in the design of the trial and contributed to all drafts of the paper. RH helped with the statistical design of the trial and contributed to all drafts of the paper. P G-F conceived and designed the trial with HA and contributed to all drafts of the paper. All authors read and approved the final manuscript.
